# HER2 Alterations in Non-Small Cell Lung Cancer: Biologico-Clinical Consequences and Interest in Therapeutic Strategies

**DOI:** 10.3390/life14010064

**Published:** 2023-12-29

**Authors:** Emma Loeffler, Julien Ancel, Véronique Dalstein, Gaëtan Deslée, Myriam Polette, Béatrice Nawrocki-Raby

**Affiliations:** 1 Université de Reims Champagne Ardenne, Inserm, UMR-S 1250 P3Cell, SFR CAP Santé, 51092 Reims, France; emma.loeffler@etudiant.univ-reims.fr (E.L.); jancel@chu-reims.fr (J.A.); vdalstein@chu-reims.fr (V.D.); gdeslee@chu-reims.fr (G.D.); myriam.polette@univ-reims.fr (M.P.); 2 CHU de Reims, Hôpital Maison-Blanche, Service de Pneumologie, 51092 Reims, France; 3 CHU de Reims, Pôle de Biologie Territoriale, Service de Pathologie, 51092 Reims, France

**Keywords:** non-small cell lung cancer, oncogenic drivers, HER2 alterations, anti-HER2 targeted therapy

## Abstract

Lung cancer stands as the first cause of death by cancer in the world. Despite the improvement in patients’ outcomes in the past decades through the development of personalized medicine approaches, a substantial portion of patients remains ineligible for targeted therapies due to the lack of a “druggable” molecular target. HER2, a receptor tyrosine kinase member of the EGFR/ErbB family, is known to show oncogenic properties. In this review, we focus on the different HER2 dysregulation mechanisms that have been observed in non-small cell lung cancer (NSCLC): gene mutation, gene amplification, protein overexpression and protein hyper-phosphorylation, the latter suggesting that HER2 dysregulation can occur independently of any molecular aberration. These HER2 alterations inevitably have consequences on tumor biology. Here, we discuss how they are not only involved in abnormal proliferation and survival of cancer cells but also potentially in increased angiogenic properties, mesenchymal features and tumor immune escape. Finally, we review the impact of these HER2 alterations in various therapeutic approaches. While standard chemotherapy and groundbreaking immunotherapy seem rather ineffective for HER2-altered NSCLCs, the development of HER2-targeted therapies such as tyrosine kinase inhibitors, anti-HER2 antibodies and especially antibody–drug conjugates could provide new hopes for patients.

## 1. Introduction

Lung cancer is currently the second most commonly diagnosed and the deadliest cancer worldwide, with more than 2.8 million new cases and an estimation of 1.8 million deaths in 2020 [1]. Lung cancer is a rather heterogeneous disease and can be divided into different histological subtypes, the two principal being small cell lung cancer (SCLC) and non-small cell lung cancer (NSCLC) [2,3]. NSCLC accounts for approximately 85% of total lung cancer cases and includes lung adenocarcinoma (LUAD), squamous cell carcinoma (LUSCC) and large cell carcinoma [2,3].

These past decades, the development of immunotherapy and targeted therapies has significantly improved the prognosis for NSCLC patients: the global 5-year survival rate increased from 10.7% in the 1970s to more than 19% in the 2010s [3]. Nonetheless, the mortality rate remains distressingly high, mainly due to late-stage diagnosis [3,4]. Moreover, a large proportion of NSCLC patients do not present a “druggable” molecular target, rendering them ineligible for targeted therapies. Indeed, NSCLCs can exhibit molecular alterations in several oncogenic drivers, such as in *Kirsten rat sarcoma* (*KRAS*) (approximately 29% of cases), *epidermal growth factor receptor* (*EGFR*) (19%), *v-RAF murine sarcoma viral oncogene homolog B* (*BRAF*) (5%), *MET* (3%), *human epidermal growth factor receptor 2* (*ERRB2*) (3%), *anaplastic lymphoma kinase* (*ALK*) (3%), *c-ROS oncogene 1* (*ROS1*) (1%) and *RET* (1%) [4]. Targeted therapies can be prescribed to patients harboring activating mutations of *EGFR*, *KRAS*, *BRAF*, *MET* or *RET* and *ALK* or *ROS1* rearrangement [5]. Regarding NSCLCs with HER2 alterations, various pharmaceutical approaches have been developed, sometimes yielding insufficient clinical results, highlighting the heterogeneity of this molecular class.

Human epidermal growth factor receptor 2 (HER2), also known as ErbB2, is a receptor tyrosine kinase encoded by the *ERBB2* proto-oncogene localized on the long arm of chromosome 17 (17q21). It is a member of the EGFR/ErbB family, which comprises HER1/EGFR, HER2, HER3 and HER4. Unlike the other members of the EGFR/ErbB family, no ligand is known for HER2. Its activation through homodimerization or heterodimerization with another ligand-bound HER family member leads to its cross-phosphorylation and activation of the tyrosine kinase catalytic domain. The activated downstream signaling pathways—mainly extracellular signal regulated kinase and mitogen activated protein kinase (ERK-MAPK), phosphatidylinositol 3-kinase and protein kinase B (PI3K-Akt) and signal transducers and activators of transcription (STAT)—are implicated in processes such as cell proliferation, survival, differentiation and migration. Consequently, HER2 alterations that result in an upregulation of its activity are involved in carcinogenesis and tumor progression [6,7,8,9].

Three main HER2 dysregulation mechanisms have been described in NSCLCs: gene mutation (1–4%), gene amplification (2–5%) and protein overexpression (2–30%) [10]. The three of them have been linked to a poorer prognosis [11,12]. However, these different types of alterations seem to originate from different mechanisms and induce different biological and clinical consequences. Hence, it has been suggested that NSCLCs with HER2 mutations, amplification and overexpression should be regarded as three distinct subtypes [10,13].

Although HER2 is an established therapeutic target in HER2-altered breast cancers, with the development and approval of monoclonal antibodies such as trastuzumab and pertuzumab as well as tyrosine kinase inhibitors (TKIs) such as lapatinib and neratinib [14], it is not the case for lung cancer. Most clinical studies evaluating HER2-targeted therapies for NSCLCs have yielded disappointing results [14,15,16], and to this day, only patients with HER2-mutated NSCLC are eligible to receive anti-HER2 drugs. 

In this review, we will describe the different types of HER2 alterations found in NSCLC. The impact of these HER2 alterations on NSCLC tumor cell biology and behavior will be discussed as well as their significance in therapeutic strategies for NSCLC patients. Ultimately, we aim to highlight new potential approaches for the management of HER2-altered NSCLCs.

## 2. HER2 Alterations

### 2.1. HER2 Gene Mutations

*HER2* gene mutations, by altering the structure of the resultant receptor, can lead to the constitutive activation of HER2 [17]. These mutations occur in approximately 1 to 4% of NSCLC cases [10,13,18,19], making them a rather rare event. This rate can reach up to 5–6% in *EGFR/KRAS/BRAF/ALK* mutation-negative patients [18,20,21]. Indeed, mutations in these oncogenic drivers and *HER2* mutations have been demonstrated to be mutually exclusive [10,13,21,22]. Moreover, *HER2* mutations have been shown to be more prevalent in patients with adenocarcinoma and in non-smokers, Asians and women [10,20,23].

*HER2* mutations almost exclusively affect the tyrosine kinase domain (TKD), with more than 90% of them being exon-20 insertions [10,21]. Among them, the most common is an in-frame 12-base-pair insertion, creating a duplication of amino acids YVMA at codon 775, also known as A775_G776insYVMA, which accounts for 50–83% of cases [10,13,19,21]. This is followed by G776delinsVC (approximately 10% of cases) and G778_P780dup (approximately 8.7% of cases) [13,19]. Other *HER2* gene mutations consist of point mutations that can affect the extracellular domain (such as S310 in exon 8), the transmembrane domain (such as V659 and G660 in exon 17) and the TKD (such as L775P in exon 19) [10,13,19]. 

Sequencing techniques like next-generation sequencing (NGS) can be employed to detect these HER2 mutations [10,13]. More cost-effective alternatives can be found in reverse-transcription polymerase chain reaction (RT-PCR) or quantitative PCR [10].

It is noteworthy that, to date, there is no substantial evidence establishing an association between *HER2* gene mutations and HER2 amplification or overexpression [10,13,24].

### 2.2. HER2 Gene Amplification

*HER2* amplification, characterized by an increase in the number of *HER2* gene copies, has been reported in approximately 2–5% of NSCLC cases [10,13,19,23], which is a rather low rate as compared to breast cancer (approximately 20–30% of cases [25]). Like *HER2* gene mutations, *HER2* gene amplification in NSCLC is more prevalent in female patients, in non-smokers and in adenocarcinoma [10,13,22].

Fluorescent in situ hybridization (FISH), the standard method for detecting *HER2* amplification, employs a *HER2* probe and a chromosome enumeration probe 17 (CEP17), which recognizes the centromere of chromosome 17 [10,13,19,23]. Generally, *HER2* gene amplification is assessed as a HER2/CEP17 ratio equal or superior to 2 [10,13,19,23]. A higher absolute number of *HER2* gene copies can be caused by chromosome 17 polysomy, typically defined as five or six copies with a HER2/CEP17 ratio inferior to 2 [10,13,19,23]. However, the prognostic or predictive value of this polysomy remains ambiguous [13,19,23,24], highlighting the importance of distinguishing patients with chromosome 17 polysomy from patients with *HER2* gene amplification. New methods to determine *HER2* gene amplification are currently under investigation, notably detection through NGS on cell-free DNA in liquid biopsies [13,26].

### 2.3. HER2 Protein Overexpression

Greater HER2 intracellular signaling activation can logically be caused by the presence of a higher number of HER2 receptors at cancer cell membranes, referred to as HER2 protein overexpression. This phenomenon has been reported to occur in 2 to 30% of NSCLC cases [10,19]. This wide range of frequency can be explained by the absence of a consensus on the method for detecting and scoring HER2 overexpression in NSCLC [10,19,22,23]. The most frequently used method is the immunohistochemistry (IHC) scoring system, in which a score ranging from 0 to 3+ is attributed to each tumor, with scores of 0–1+ being defined as negative, 2+ as weak to moderate in ≥10% of tumor cells and 3+ as strong in ≥10% of tumor cells [10,13]. With that distinction, IHC 2+ represent approximately 24% and IHC 3+ 3–10% of NSCLC cases [13,19]. The other scoring method is the H-SCORE, in which staining intensity is multiplied by the percentage of positive tumor cells, giving a range of 0 to 300: scores of <100 are defined as negative, ≥100–200 as moderate and ≥200 as high [13,19]. More recently, the detection of HER2 overexpression at the mRNA level (with the use of RT-qPCR, for example) has been suggested as an alternative [10,13,22]. Notably, using a cut-off value of 1.8 for the ratio between lung tumors and normal tissues, Brabender et al. observed that high HER2 mRNA levels were associated with a poorer prognosis [27].

To this day, whether there is an association between *HER2* gene amplification and HER2 protein overexpression in NSCLCs remains unclear [10,19]. Indeed, a correlation between HER2 protein expression determined via IHC scoring and HER2 gene amplification determined via FISH assay has been found by Bunn et al. in NSCLC cell lines [28]. However, cases of IHC-negative but FISH-positive tumors have also been reported [19,22].

### 2.4. HER2 Protein Hyper-Phosphorylation

HER2 gene mutation, gene amplification and protein overexpression might not be the only mechanisms of HER2 signaling dysregulation involved in NSCLCs. Indeed, some studies have taken an interest in directly examining the activated form of HER2, phospho-HER2 (pHER2) [29,30,31,32]. Unlike the three molecular types of alteration described previously, hyper-phosphorylation of HER2 constitutes a phenotypic alteration that can also reflect a greater activity of HER2.

The percentage of pHER2-positive NSCLC patients is rather complicated to assess since there is no standard method of detection. Indeed, while multiple residues are phosphorylated when HER2 is activated, only one of these residues is recognized (depending on the anti-pHER2 antibody), therefore potentially leading to underestimation of the pHER2 rate [29]. It has also been shown that using phosphoproteins as biomarkers in human tumors is a challenging process, given the instability of phosphorylations and their rapid tendency to dephosphorylate [33].

In a panel of 249 NSCLC cases, Suzuki et al. observed that 83 (33%) were positive for pHER2^Y1221/1222^ [29]. Among these 83 pHER2-positive tumors, it is worthy to note that 64 (77%) did not overexpress HER2, 16 (19%) did not present HER2 amplification and 54 (65%) did not present HER2 mutations [29]. Scrima et al. took interest in pHER2^Y1248^ and found that among 114 NSCLC tumors, 41 (36%) had high levels of pHER2, in similar proportions in LUAD (39%) and LUSCC (32%), as well as in early stage (36%) and advanced disease (39%) [30]. These high levels of pHER2 were not correlated with overexpression or amplification of HER2 [30]. Indeed, 27/40 (67%) of pHER2-positive tumors did not overexpress HER2 and 30/34 (88%) did not present HER2 amplification [30]. Finally, in a panel of 45 NSCLC cases, we observed that 18 (40%) had high levels of pHER2^Y1248^, which was not associated with HER2 overexpression [31]. We found that this hyper-phosphorylation of HER2 was a consequence of the loss in tumor suppressor fragile histidine triad (FHIT) [31] and that patients displaying a FHIT^low^/pHER2^high^ phenotype could present a poorer prognosis [32]. Consistent with these results, Scrima et al. observed that pHER2 positivity was significantly associated with reduced overall survival (OS) and disease-free survival (DFS) in patients with early-stage NSCLC [30]. Interestingly, similar observations have been made in other cancers; indeed, several studies have associated HER2 phosphorylation with poorer prognosis and survival in breast cancer [34,35,36]. However, Suzuki et al. found that among NSCLC patients presenting *HER2* mutations, pHER2 positivity was associated with a better outcome [29].

Altogether, these data suggest that HER2 activation through phosphorylation may be a phenotypic change independent of genetic alterations. Moreover, HER2 hyper-phosphorylation might hold a prognostic and/or predictive value, which highlights the relevance of screening patients for their pHER2 profile rather than solely looking for molecular aberrations.

To conclude, HER2 can be altered in many ways in NSCLCs, and the complex interrelationships between the different types of alterations remain to be fully elucidated. Given the different clinical characteristics specifically associated with these alterations, it is crucial to correctly identify them with standardized methods of detection and interpretation of results. The key points regarding the different types of HER2 alterations in NSCLCs and their detection are compiled in Table 1.

## 3. Biological Consequences of HER2 Alterations

Since HER2 is upstream of various intracellular signaling pathways, dysregulation of its activity resulting from the previously described mechanisms has consequences in tumor cell biology and behavior. Moreover, it has been shown that HER2 is the preferred dimerization partner for all the other members of the EGFR/ErbB family [37]. Consequently, HER2 alterations might possibly interfere with EGFR-, HER3- and HER4-related signaling as well. Overall, intracellular signaling pathways induced by the EGFR/ErbB family promote processes that are favorable to tumor progression, with the more described ones being survival and proliferation. In this part, we review the relationship between HER2 alterations and hypoxia/angiogenesis, epithelial-to-mesenchymal transition (EMT) and tumor immune escape in NSCLCs.

### 3.1. Hypoxia and Angiogenesis

Hypoxia is defined as a decreased availability of oxygen in tissues and has been shown to serve as a favorable factor in tumor progression, notably by promoting angiogenesis [38]. The term “angiogenesis” refers to the formation of new blood vessels from pre-existing vasculature and is one of the hallmarks of cancer [39]. Indeed, tumor growth depends upon a sufficient supply of oxygen and nutrients. To this end, cancer cells acquire the ability to induce blood vessel development, mainly through secretion of pro-angiogenic factors such as vascular endothelial growth factor (VEGF), basic fibroblast growth factor, angiopoietin 1 and 2, platelet-derived growth factor, etc. [39,40].

In NSCLCs, tumor hypoxia and expression of pro-angiogenic markers have been correlated with a worse prognosis [40,41]. Consequently, several strategies are currently in development to target hypoxia and angiogenesis in lung cancer [42]. Bevacizumab, an anti-VEGF antibody, has even been approved for the treatment of NSCLCs [43].

Given its oncogenic properties and the intracellular signaling pathways that are regulated upon its activation, several studies have taken interest in looking at the role of HER2 in the promotion of angiogenesis. Interestingly, Whelan et al. observed in transgenic mice that Neu+ tumors (Neu being the rodent homologue of HER2) expressed higher levels of hypoxia-induced factor 1α (HIF-1α) [44], a transcription factor notably responsible for the activation of VEGF expression in response to hypoxia [38]. They also suggested that hypoxia and HER2 overexpression lead to similarities in cell phenotypes and gene expression profiles [44]. Consistent with these results, several studies reported a link between amplification or overexpression of HER2 and higher levels of HIF-1α, VEGF or angiogenesis in human breast cancer models [45,46,47,48]. Studies conducted in human gastric cancer found higher microvessel density in HER2 positive tumors but without achieving significant results [49,50]. Finally, Zhang et al. showed that the use of two anti-HER2 antibodies, chA21 and trastuzumab, lead to greater inhibition of angiogenesis in human ovarian cancer [51].

These different findings suggest that dysregulation of HER2 could participate in the promotion of angiogenesis in human cancers. However, although it seems plausible that HER2 alterations could lead to similar consequences in NSCLC, more data are needed to extend those observations to lung cancer.

### 3.2. Epithelial-to-Mesenchymal Transition

EMT is a complex molecular process through which cells lose their epithelial characteristics to acquire mesenchymal features. This process is characterized by a downregulation of epithelial markers (such as E-cadherin) and an upregulation of mesenchymal markers (such as vimentin, fibronectin and N-cadherin), leading to a loss in intercellular junctions and apico-basal polarity in favor of a contractile and motile phenotype [52,53]. EMT is involved in diverse physiological processes, such as embryonic development and wound healing, as well as in pathological processes, such as cancer [52,53]. Indeed, EMT provides tumor cells with enhanced migratory and invasive capacities, survival, angiogenic potential, immune evasion and treatment resistance, therefore promoting tumor progression and metastasis dissemination [54].

In the case of NSCLCs, mesenchymal attributes are frequently found in both LUAD and LUSCC [54]. Interestingly, it has been suggested that EMT might hold a predictive value for early-stage NSCLC patients, given that mesenchymal features seem to be associated with a worse prognosis [54]. For example, it has been shown that vimentin expression in NSCLC tumors correlates with the occurrence of metastases [55].

HER2 controls various intracellular pathways that can be involved in cancer progression by driving tumor cell motility. Indeed, it has been well documented that the activation of ERK-MAPK and PI3K-Akt pathways participate in cancer cell migration and invasion [56,57,58], notably by regulating EMT [59,60]. For example, it is clearly established that transforming growth factor β induces EMT through the ERK-MAPK and PI3K-Akt pathways in epithelial cells [61,62]. Since the ERK-MAPK and PI3K-Akt pathways are also controlled by HER2, it is of interest to investigate the impact of HER2 alterations regarding EMT in NSCLC.

Li et al. found that miR-331-3p, which is downregulated in NSCLC tumors, is able to control EMT, migration and metastasis dissemination of NSCLC cells through the Rac1/PAK1/β-catenin pathway by directly targeting VAV2 and HER2 expression [63]. In presence of miR-331-3p, the transfection of a mimic to overexpress HER2 leads to partial restoration of EMT and invasion [63]. In accordance with these results, we observed that the hyper-phosphorylation of HER2 induced by an invalidation of FHIT in NSCLC cell lines was concomitant with an upregulation of vimentin, a downregulation of E-cadherin and increased cell invasion [31]. The use of two different anti-HER2 targeted therapies on FHIT^low^ cells lead to the inhibition of vimentin induction and re-localization of E-cadherin to the cell membrane in association with a reduction in invasive capacities [31].

These different findings suggest that HER2 could play an active role in the onset of EMT in NSCLC cells and that HER2 alterations might be associated with more mesenchymal features in NSCLC tumors. Supporting these data, the promotion of tumor cell invasiveness and EMT in cancer cells by the EGFR/ErbB family has previously been reviewed [64,65] and observed in various cancers. For example, Jeon et al. showed that HER2 overexpression in breast cancer cells promotes the expression of fibronectin, which increases cell invasion capacities [66]. Liu et al. reported that knockdown of HER2 in gastric cancer cells leads to decreased invasiveness and downregulation of mesenchymal markers such as N-cadherin and Twist [67].

### 3.3. Tumor Immune Escape

According to the tumor immuno-editing theory, cancer cells can be specifically recognized by the immune system, notably through the presence of abnormal proteins at their surface called neoantigens, and therefore be eliminated by T cells [68,69]. However, by developing various immune escape mechanisms, tumor cells are able to progressively reduce the efficiency of this antitumor immune response and evade surveillance [68,69]. In NSCLC, many of these mechanisms have been identified in the early stages as well as in the more advanced ones [69]. Interestingly, it has been reported that breast tumors with HER2 amplification seem to present a “cold” tumor microenvironment, with less tumor-infiltrating lymphocytes [68]. Moreover, higher expression of interferon-stimulated gene and lymphocyte infiltration have been observed after targeting HER2 in cancer treatment studies [70]. These different findings suggest that the dysregulation of HER2 might play an active role in tumor immune evasion, and several studies have taken interest in looking for the molecular mechanisms behind this phenomenon.

The cGAS-STING pathway is a cellular damage surveillance system able to sense aberrant cytosolic DNA fragments, which are rather frequent in tumor cells due to genomic instability [70]. Its activation leads to increased type I interferon expression and cytokine production, resulting in cancer cell senescence or apoptosis [70]. Interestingly, Wu et al. found that HER2 is able to inhibit this innate antitumor immune mechanism by recruiting Akt to STING, leading to the impairment of the STING signalosome assembly [70].

Major histocompatibility complex (MHC) class I molecules play an essential role in antitumor immunity. Indeed, they can bind with small (8–10 amino acids long) peptide antigens, such as neoantigens, in order to present them at the cell surface for recognition by cytotoxic CD8+ T lymphocytes (CTL) [68,71]. However, it has been reported that HER2 overexpression is associated with reduced levels of major histocompatibility complex (HMC) class I molecules in several models, including murine fibroblasts [72], mammary carcinoma [73], human melanoma [74], esophageal squamous cell carcinoma (ESCC) [75] and breast cancer [76]. The invalidation of HER2 in ESCC and breast cancer cell lines leads to an upregulation of MHC class I molecules [75,76,77], resulting in increased CTL recognition [75]. These results suggest that HER2 might play an active role in the downregulation of MHC class I molecule expression and therefore impair neoantigen presentation and CTL recognition.

Interestingly, through a RNA-Sequencing analysis of 12 NSCLC cases, we found that genes that were downregulated in tumors with an FHIT^low^/pHER2^high^ phenotype were functionally enriched for immune response [32]. There was notably a downregulation of MHC class II molecules [32], which are essential for presenting antigens to CD4+ T lymphocytes.

These findings suggest that HER2 alterations could be involved in escaping antitumor immunity. However, since not much data are available for lung cancer, further studies are needed to fully assert whether HER2 does play a role in tumor immune evasion in NSCLCs as well as the underlying molecular mechanisms that are involved.

To conclude, in addition to the well-described pro-proliferative and pro-survival effects of HER2 dysregulation, it seems that HER2 alterations could also be involved in the promotion of hypoxia/angiogenesis, EMT and tumor immune escape based on findings made in various cancer models. To support these different individual observations, it is relevant to point out that hypoxia, EMT and immune evasion are processes that are considerably interconnected. Indeed, it has been reported that hypoxia in the tumor microenvironment (TME) can drive EMT in cancer cells and tumor immune escape [78,79,80]. Moreover, there are many mechanisms through which cancer cells undergoing EMT can modulate the TME and therefore regulate antitumor immunity [81,82], notably in lung cancer [83,84]. Even if the different observations reported in this section still need to be validated in HER2-altered lung cancer, they do seem plausible since HER2 signaling could act as one of the crossroads between these different processes.

## 4. Clinical Consequences of HER2 Alterations and Therapeutic Strategies

Given that HER2 alterations can have consequences on cancer cell behavior and tumor characteristics, it is relevant to investigate their impact on patients’ responses to treatments. As previously mentioned, the distinct subgroups of HER2 alterations described in NSCLC have been associated with specific clinical patterns [11,21,32]. In this part, we therefore review the clinical benefit provided by chemotherapy, immunotherapy and HER2-targeted therapies in the case of HER2-altered NSCLCs.

### 4.1. HER2 Status and Response to Chemotherapy

The common first-line treatment for NSCLCs without any oncogenic driver relies on platinum-based chemotherapy until progression or limiting toxicities [85]. Without molecular alteration, chemotherapy provides a median PFS of 4.4 months and an OS of 13.9 months based on the PARAMOUNT study [86].

Chemotherapy efficacy has been specifically explored in the context of HER2 alterations with various and inconstant results. In a cohort of 53 NSCLCs (among 184 cases), Cappuzzo et al. evaluated the impact of HER2 amplification on chemotherapy and reported no association between the HER2 gene copy number and the response to first-line chemotherapy [87]. Furthermore, despite the absence of a clear association between HER2 status and chemotherapy responsiveness in its cohort, Kuyama et al. also reported a worsened prognosis in 23 patients with HER2 FISH-positive NSCLC who underwent radio-chemotherapy [88]. This observation was further corroborated in an external cohort study [89]. More recently, Wang et al. explored the efficacy of first-line pemetrexed-based chemotherapy for patients with advanced HER2-mutant lung adenocarcinomas [90]: 29 (5.1%) of patients with HER2 mutations were identified with a shorter median PFS (assessed at 5.1 months) in comparison with the ALK/ROS1 group (*p* = 0.004). Molecular subclass even impacted the prognosis, with a shorter median PFS of 4.2 months for the most common HER2 mutations (i.e., exon-20 mutation A775_G776insYVMA).

Considering these findings, it appears that while chemotherapy may not provide optimal outcomes, it remains a standard treatment due to the lack of more suitable alternatives and occasional individual responses.

### 4.2. HER2 Status and Reponse to Immunotherapy

Over the last few decades, a significant breakthrough in lung cancer treatment has been the use and effectiveness of immune checkpoint inhibitors (ICIs). Initially, they were employed for metastatic stages [91], then as consolidation therapy after chemo-radiation for locally advanced NSCLCs [92], and more recently in the perioperative context for early stages [93]. All these clinical trials and drug indications are now excluding common oncogenic drivers such as EGFR or ALK alterations based on non-efficacy and even higher risk of toxicities [94]. Here, we review clinical trials reporting clinical outcomes for NSCLCs with HER2 alterations.

Firstly, the Memorial Sloan Kettering Cancer Center (MSKCC) reported a cohort of 26 patients treated with ICIs among 122 HER2-mutant NSCLCs [95]. Interestingly, PD-L1 expression was lower in the HER2-mutants, with 77% of the cases being PD-L1 negative (*p* = 0.006). However, the tumor mutational burden (TMB), potentially related to tumor immune sensibility, was similar to an unselected cohort. In patients treated with anti-PD-(L)1, objective response rate (ORR) was only of 12%, including three partial responses and eight stable diseases, resulting in a shorter median PFS of 1.9 months. These findings were in concordance with data from the MD Anderson Lung Cancer Center, where 16 patients with HER2-mutant NSCLCs were treated with ICIs, resulting in one partial response and two cases of stable disease. The median PFS in this group was 1.8 months [96]. Another retrospective trial investigated the interest of immunotherapy in 511 patients with identified oncogenic drivers, including 29 HER2-mutant NSCLCs [97]. The results were broadly similar, with a median PD-L1 expression of 0%, an ORR of 7% and a median PFS of 2.5 months. Exploratory analyses reported a potential of ICIs for smoker patients (vs non-smokers) with HER2-mutant NSCLCs, with a median PFS reaching 3.4 months. Moreover, the clinical efficacy of ICIs in 18 HER2-amplified NSCLCs was recently reported at the 2022 ASCO congress [98]. Sixty-nine percent of tumors were PD-L1 negatives with a median TMB of 9.2 mutations/Mb. The median PFS and OS were 11 weeks and 2 months, respectively, also illustrating a minimal response to ICIs. Still retrospectively, the French Lung Cancer Group assessed clinical outcomes for patients with some molecular alterations, including 23 HER2 mutants, treated with ICIs [99]. The ORR was 27.3% for these patients, mainly treated in the second or third line with nivolumab. The median PFS remained shorter, estimated at 2.2 months but with a median duration of response (DOR) of 15.2 months for the few responders.

All these results derived from retrospective studies present consistent data indicating that ICIs are not a suitable option for patients with altered-HER2 NSCLCs, although they might still be considered in the absence of alternative options. Nonetheless, the occurrence of prolonged and profound responses suggests that certain patients might still benefit from immunotherapy, highlighting the necessity for more accurate predictive markers of efficacy. Interestingly, by using the tumor immune dysfunction and exclusion model on LUAD and LUSCC cohorts from the TCGA to predict their response to ICIs, we showed that patients displaying a FHIT^low^/pHER2^high^ phenotype might not benefit from immunotherapy [32]. Moreover, to date, only a few trials are specifically exploring the potential of ICIs for HER2-mutant NSCLCs. For example, NCT04144569 proposes to combine anti-PD-1 with pyrotinib (an anti-HER2 TKI described below) for NSCLCs with HER2 insertion mutations and progressing after a chemotherapy. This trial is still recruiting participants.

### 4.3. HER2-Targeted Therapies

All these disappointing results from conventional therapies and the poorer prognosis related to HER2-altered NSCLCs strengthen the need for effective HER2-targeted drugs in clinical practice. Among them, two major classes are being developed: TKIs and HER2 antibodies, with quite promising results for some of them in NSCLCs harboring HER2 alterations (Table 2). However, and in contrast to breast and gastric cancers, these drugs remain not yet considered as a standard of care in HER2-altered NSCLCs.

#### 4.3.1. Tyrosine Kinase Inhibitors

TKIs are antagonist molecules that bind to tyrosine kinase domains in order to disrupt the activation of downstream signaling pathways. Various TKIs have been developed to target HER2, with respective profiles of efficacy and toxicity.

Afatinib is a non-selective TKI that irreversibly inhibits the EGFR/ErbB family. It was FDA-approved to treat metastatic NSCLCs harboring EGFR mutations before osimertinib’s approval [115]. Afatinib was thus evaluated in the context of HER2-mutant NSCLCs in three phase II trials, all with negative results. De Grève et al. did not report any response in seven patients treated with afatinib, with a median PFS of 17 weeks in this heavily pre-treated cohort [116]. Afatinib exhibited no better efficacy in a larger single-arm trial of 28 patients, with an ORR of 19% and a median PFS of 2.9. months [117]. The last trial, named NICHE, included 13 patients and reported only one response and a median PFS of 15.9 weeks [100]. Afatinib thus does not appear to be a relevant option for HER2-mutant NSCLCs.

Neratinib is also an irreversible non-selective TKI that inhibits the EGFR/ErbB family. Its activity on HER2-altered NSCLCs was investigated into two phase II trials. The SUMMIT study was a basket trial enrolling 26 HER2-mutant NSCLCs among a multi-histology cohort of 141 patients treated with neratinib [103]. Unfortunately, only two responses were noticed (ORR = 3.8) with a minimal efficacy, illustrated by a median PFS of 5.5 months allowed by a disease control rate (DCR) of 42.3%. The second trial, named PUMA-NER-4201, combined neratinib with temsirolimus (an mTOR inhibitor) in 60 patients with NSCLCs harboring HER2 mutations [102]. Compared to neratinib alone, the combination allowed improvement in the ORR from 0% to 19%, with limited efficacy despite some few prolonged responses and a median PFS of 4.1 months (vs. 3 months for the neratinib arm).

Dacomitinib, another TKI, has a similar profile of activity to neratinib. It was evaluated in a phase II trial including 30 patients distributed into 26 HER2-mutant (with 25 exon-20 insertions) and 4 HER2-amplified NSCLCs [101]. No signal of efficacy was noticed among the HER2-amplified NSCLCs. Some signal was reported among the HER2-mutant specimens with three prolonged responses. However, the ORR was only 12% with a median PFS of 3 months. Safety was also a real concern, with 23% having grade 3–4 diarrhea.

Given the limited efficacy and the risks of toxicity of the different inhibitors described above, more selective TKIs have been developed in the last decades in order to provide more efficient anti-HER2 targeted therapies.

Poziotinib was first evaluated in a restricted cohort of 12 HER2-mutant NSCLCs [118]. A signal of activity was reported with an ORR of 42% and a median PFS of 5.6 months despite a skin toxicity leading to a dose reduction for 67% of patients. A similar cohort of 13 patients reported an ORR of 50% at 8 weeks with a DCR of 83% [119]. The ZENITH-20 trial managed to enroll 90 patients and confirmed this relative efficacy with a median PFS of 5.5 months, an ORR of 27.8% and a DCR of 70.0% [120]. More recently, a cohort of 30 patients with NSCLCs presenting HER2 exon-20 insertions and treated with poziotinib was published [121]. Consistently, the ORR was 27% with a median PFS of 5.5 months and a median OS of 15 months despite a heavily pre-treated cohort (53% were in the second line or more). These three prospective clinical trials were pooled in a recent meta-analysis published in 2022, confirming the antitumor activity in HER2 exon-20 mutant NSCLC patients [122].

Pyrotinib is another anti-HER2 TKI that has been evaluated in HER2-mutant NSCLCs. A phase I-II trial of 15 patients treated with pyrotinib thus demonstrated the higher response rate observed among various anti-HER2 TKIs, with an ORR of 53.3% and a median PFS of 6.4 months [123]. It is worthy to note that the safety profile was good and without any grade 3–5 or dose reduction reported. This promising activity was confirmed in a larger phase II trial of 60 patients treated with pyrotinib in monotherapy [106]. The assessed ORR was 30.0%, and both the median DOR and median PFS were 6.9 months. Another external phase II trial with 78 patients observed similar PFSs and ORRs [107]. Finally, pyrotinib efficacy was also evaluated in a cohort of 27 patients with HER2-amplified NSCLCs, exhibiting antitumor activity with an ORR and a median PFS of 22.2% and 6.3 months, respectively [124].

Other anti-HER2 TKIs include tarloxotinib, a hypoxia-related prodrug with relative efficacy that is no longer developed as monotherapy [125], as well as mobocertinib and tucatinib.

Mobocertinib (TAK-788) is an irreversible TKI designed to target HER2 exon-20 insertion mutations in NSCLCs [126]. Mobocertinib showed high selectivity and strong inhibition in various models, including allograft and patient-derived xenograft models. In mouse models, it induced sustained complete responses for those with certain HER2 exon-20 mutations, while others showed partial responses. These findings support ongoing clinical trials for mobocertinib (NCT02716116) and suggest that lung adenocarcinoma patients with a HER2 exon-20 A775_G776insYVMA mutation could potentially be treated with a combination of mobocertinib and T-DM1 [127].

Tucatinib is another highly selective anti-HER2 TKI targeting the HER2 tyrosine kinase domain. When comparing tucatinib and a placebo in combination with trastuzumab and capecitabine to treat HER2-positive breast cancer patients, the HER2 CLIMB study demonstrated that both the PFS and OS were significantly longer in the tucatinib group [128]. Notably, tucatinib exhibits favorable penetration into the brain. In 2020, the FDA approved this regimen for locally advanced unresectable or metastatic HER2-positive breast cancer. However, the effectiveness of tucatinib against HER2-mutated NSCLCs remains undetermined and is currently under evaluation in ongoing trials (NCT04579380).

Finally, new selective HER2-targeting TKIs that are currently in development, such as zongertinib (BI 1810631) [129,130] and ELVN-002 [131,132], were presented at recent international congresses, with only preliminary and partially published results to date.

#### 4.3.2. Antibodies

Anti-HER2 antibodies, such as trastuzumab or pertuzumab, have long been based on blocking antibodies. Recent advances also resulted in alternative compounds such as antibody–drug conjugates (ADCs), with promising results for patients with HER2-positive NSCLC.

##### Monoclonal Antibodies (mAbs)

Trastuzumab is a humanized murine monoclonal IgG1 antibody that binds to the HER2 extracellular domain, inhibiting its dimerization. In the HOT1303-B trial, trastuzumab monotherapy did not elicit any response among patients classified as HER2-positive NSCLCs (due to overexpression or mutation) [133]. Therefore, the combination of trastuzumab with chemotherapy has also been evaluated in patients with HER2-positive NSCLC. Mainly based on HER2 overexpression, trastuzumab has been associated with either cisplatin and gemcitabine [16], docetaxel [134], paclitaxel [135], or carboplatin and paclitaxel [15]. All these trials demonstrated disappointing efficacy, with an ORR ranging from 0% with docetaxel to 36% with a gemcitabine–cisplatin combination. One randomized trial compared a gemcitabine–cisplatin regimen +/− trastuzumab in untreated patients with HER2-overexpressing NSCLC. No improvement was reported between the chemotherapy arm (ORR of 41% and median PFS of 7.0 months) and the trastuzumab + chemotherapy experimental arm (ORR of 36% and median PFS of 6.1 months).

Another monoclonal antibody targeting HER2 and inhibiting its dimerization is pertuzumab. The most relevant trial exploring pertuzumab use in HER2-mutant NSCLC patients was the IFCT-1703 R2D2 trial [109]. This non-randomized phase II trial enrolled 45 patients who had progressed after at least one platinum-based first-line treatment. Patients were treated with a combination of pertuzumab, trastuzumab and docetaxel administered every 3 weeks until disease progression. The ORR was 29%, with 58% of patients achieving a stable disease and a median PFS of 6.8 months, suggesting a potential efficiency for this triple therapy.

##### Antibody–Drug Conjugates (ADCs)

To date, two anti-HER2 ADCs have been tested in NSCLCs: trastuzumab–emtansine (T-DM1) and trastuzumab–deruxtecan (T-DXd), both with available results.

Trastuzumab–emtansine is an ADC based on trastuzumab loaded with DM-1, a cytotoxic microtubule inhibitor. To evaluate its efficacy, a trial enrolled 49 patients with HER2 overexpression, but the results were very limited, with no responses among HER2 2+ patients and only four partial responses for HER2 3+ patients [111]. HER2-mutant NSCLCs were more extensively explored in three phase II trials [110,136,137]. All these trials included a majority of patients with HER2 exon-20 insertion NSCLC and demonstrated limited effectiveness. The ORR ranged from 6.7% to 44% and the median PFS from 2.0 months to 5.0 months. However, these mixed results showed potential activity and suggested T-DM1 as a potential option for patients with NSCLC with HER2 exon-20 insertion.

More recently, trastuzumab–deruxtecan (DS-8201, i.e., Enhertu) has emerged as a relevant ADC to target HER2-mutant NSCLCs [138,139]. T-DXd is an ADC composed of trastuzumab loaded with deruxtecan, a topoisomerase I inhibitor. T-DXd is currently revolutionizing the treatment of the HER2 molecular sub-class in breast carcinoma and is also the only FDA-approved targeted therapy for NSCLC patients with HER2 mutations. In the DESTINY-Lung04 trial, T-DXd was evaluated in two conditions: NSCLCs overexpressing HER2 (defined by IHC score ≥ 2+) and HER2-mutant NSCLCs. Forty-nine patients with HER2-overexpressing metastatic NSCLCs received T-DXd at 6.4 mg/kg every 3 weeks in a non-randomized phase II trial [112]. Among these patients, 10 (20.4%) and 39 (79.6%) had a HER2 IHC score of 3+ and 2+, respectively. In this interim analysis, despite 34.7% of central nervous system metastasis and heavily pre-treated patients (with a median number of prior regimens of 3), ORR was of 24.5% including 1 complete response and 11 partial responses. The median PFS was 5.4 months with a median DOR of 6.0 months. The toxicity remained manageable. However, interstitial lung diseases (ILDs) induced by T-DXd were observed in eight cases (16.3%), therefore requiring specific monitoring and clinical vigilance. T-DXd was also investigated in HER2-mutant NSCLCs in a multi-center, international, non-randomized phase II trial published in 2022 [113]. Ninety-one patients with a metastatic NSCLC harboring HER2 mutations were treated with T-DXd at 6.4 mg/kg every 3 weeks. With a median follow-up of 13.1 months, the authors reported an ORR of 55% with a median DOR, PFS and OS of 9.3, 8.2 and 17.8 months, respectively. The safety was similar to that previously published, with an adjudicated drug-related ILD observed in 26% of patients and two related deaths. Responses were independent of the HER2 mutation subtypes, HER2 expression or amplification. These data were confirmed by recent results exposed at the IASLC WCLC 2023: T-DXd provided a median PFS of 9.9 months at 5.4mg/kg and 15.4 months at 6.4mg/kg, with a median DOR of 16.8 months at 5.4 mg/kg (not reached for the 6.4 mg/kg dose) [114].

In addition to anti-HER2 TKIs and antibodies, recent technological advancements enabled the development of complementary approaches. One notable example is SAR443216, a tri-specific T-cell engager designed to target human HER2-expressing cancer cells. SAR443216 comprises one HER2 binding domain, one CD3 binding domain for T-cell binding and activation and a CD28 binding domain that offers a co-stimulatory signal to T cells. It is currently being evaluated in a phase I/Ib trial by Dumbrava et al. [140].

To conclude this section on anti-HER2 targeted therapies in NSCLC, a meta-analysis pooled data from 32 clinical trials, which included 958 patients treated with various anti-HER2 therapies ranging from TKIs to ADCs [141]. The ORRs related to ADCs were approximatively 60%, whereas they ranged from 16% to 26% for TKIs and monoclonal antibodies. Disease control rates were also higher for ADCs, estimated at 87% and falling to 31–59% for other treatment options. Median PFSs were consistent, ranging from 2.65 to 5.51 months for HER2-TKIs and monoclonal antibodies against 12.04 months for ADCs. Excluding ADCs, poziotinib and pyrotinib showed a stronger signal of clinical activity in the subgroup analysis. To date, T-DXd, poziotinib and pyrotinib can therefore be considered as relevant treatments for NSCLC patients with HER2 molecular alterations.

Still underexplored, hyper-phosphorylated HER2 NSCLCs might also be considered as promising candidates for anti-HER2 targeted therapies. Indeed, our research findings have demonstrated that cancer cells from NSCLC patients harboring a FHIT^low^/pHER2^high^ phenotype exhibited greater responsiveness to anti-HER2 targeted therapies (such as trastuzumab and tucatinib) compared to other phenotypes [31]. These findings are supported by several studies conducted in breast cancer [142,143,144,145,146]. For example, Burguin et al. showed that HER2−/pHER2^Y877^+ breast cancer cells were sensitive to trastuzumab, therefore suggesting that patients without any molecular alterations in HER2 but a pHER2+ profile might benefit from an anti-HER2 targeted therapy [146].

## 5. Conclusions

Lung cancer remains the deadliest cancer worldwide, and the development of novel therapeutic approaches is of utmost importance, especially for patients with driver mutations who may not be eligible for targeted therapies. In this regard, what we provide here is a synthetic overview of the current knowledge on HER2 aberrations in NSCLCs and their impact on tumors’ biological features as well as on treatment strategies for patients.

Three types of HER2 alterations are rather well described in the literature on NSCLCs: gene mutation, gene amplification and protein overexpression, all of which lead to a greater activity of HER2 in cancers cells. These three different alterations have been associated with specific clinical patterns and, as suggested in several papers, should therefore be regarded as three distinct subtypes. In that sense, even more sensitive and specific methods of detection for all three of these aberrations are currently under development. In this review, we also discuss the existence of another mechanism of HER2 signaling dysregulation, namely HER2 hyper-phosphorylation, which constitutes a phenotypic change rather than a molecular one. Several studies suggest the relevance of screening patients for their pHER2 phenotype, notably in terms of predicting patients’ outcomes or responses to therapies.

Considering the different signaling pathways that are downstream of HER2, dysregulation of its activity through the previously mentioned mechanisms can have various biological consequences that contribute to tumor progression. In addition to the well-described pro-proliferative and pro-survival effects of HER2 activation, this review delved into the potential involvement of HER2 alterations in promoting hypoxia/angiogenesis, EMT and tumor immune escape.

Finally, given the potential implications of HER2 alterations in cancer cell behavior and overall tumor biology, it is crucial to explore the impact of HER2 dysregulation on therapeutic strategies. Several studies report a limited efficacy of chemotherapy as well as immunotherapy for HER2-altered NSCLCs. To address this challenge and align with the growing interest in personalized cancer treatment, various anti-HER2 targeted therapies are currently under development and testing. Among them, two non-selective TKIs, poziotinib and pyrotinib, are returning rather promising results. However, the most hopeful approach for patients involves the use of an anti-HER2 antibody combined with a cytotoxic agent. Indeed, while simple monoclonal antibodies have shown limited effects, T-DXd, an ADC currently revolutionizing breast cancer treatment, is establishing itself as the most relevant anti-HER2 therapy in NSCLCs to date. Nonetheless, a better understanding of the intricate molecular mechanisms involved in HER2-altered cancer cells in relation to tumor progression, angiogenesis, immune escape and treatment resistance is still necessary to develop even more efficient therapeutic strategies.

## Figures and Tables

**Table 1:** Types of HER2 alterations found in NSCLCs and their detection.

Type of Alteration	Frequency	Method of Detection	Interpretation of Results
*HER2* gene mutation	1–4%	NGS * RT-(q)PCR	Presence of A775_G776insYVMA (50–83% of cases), G776delinsVC (10%), G778_P780dup (8.7%), etc.
*HER2* gene amplification	2–5%	FISH * NGS	HER2/CEP17 ≥ 2: positive (If multiple *HER2* copies but *HER2/CEP17* < 2: chromosome 17 polysomy)
HER2 protein overexpression	2–30%	IHC * RT-qPCR	IHC 0–1+: negative IHC 2+: weak to moderate in ≥10% of tumor cells IHC 3+: strong in ≥10% of tumor cells
HER2 protein hyper-phosphorylation	30–40%	IHC	No standard (Detection of Y1221/1222, Y1248, etc.)

* Standard method. NGS: next-generation sequencing; RT: reverse transcription; (q)PCR: (quantitative) polymerase chain reaction; FISH: fluorescent in situ hybridization; IHC: immunohistochemistry; CEP17: chromosome enumeration probe 17.

**Table 2:** Pivotal studies on selected anti-HER2 drugs in NSCLCs with HER2 alterations.

Trial	Drug	Population	n	Phase	Line	Efficacy				Safety G3+ TRAE	Reference
						ORR	mPFS (Months)	mDoR (Months)	mOS (Months)		
HER2 TKIs											
NICHE	Afatinib	HER2 mutation (exon-20 insertion)	13	II	≥2L	53.8%	3.9	NR	14.0	<10%	[100]
MG Kris et al.	Dacomitinib	HER mutations/amplification	30	II	≥2L	12% (3/26 HER2 mutated), 0% (HER2 amplified)	3	NR	9.0	NR	[101]
PUMA-NER-4201	Neratinib +/− temsirolimus	HER mutations	62	II	≥2L	0% (neratinib) 19% (8/43 combined)	3 (neratinib) 4.1 (combined)	NR	10.0 (neratinib) 15.8 (combined)	G3 diarrhea (12% neratinib, 14% combined)	[102]
SUMMIT	Neratinib	HER2/3 mutations	26	II	≥2L	4% (1/26)	5.5	NR	NR	G3 diarrhea (22%)	[103]
ZENITH-20 (cohort 2)	Poziotinib (16 mg QD)	HER2 mutation (exon-20 insertion)	90	II	≥2L	27.8%	5.5	5.1	NR	G3 rash (48.9%), G3 diarrhea (25.6%), G3 stomatitis (24.4%)	[104]
ZENITH-20 (cohort 4)	Poziotinib (16 mg QD or 8 mg QD)	HER2 mutation (exon-20 insertion)	80	II	1L	39%	5.6	5.7	NR	G3 rash (QD, 45%; BID, 39%), stomatitis (QD, 21%; BID, 15%), diarrhea (QD, 15%; BID, 21%)	[105]
Zhou et al.	Pyrotinib	HER2 mutation	60	II	≥2L	30%	6.9	6.9	14.4	G3 or G4 (28.3%), G3 diarrhea (20%)	[106]
Song et al.	Pyrotinib	HER2 mutation	78	II	≥1L	19.2%	5.6	9.9	10.5	20.5%	[107]
PATHER2	Pyrotinib + apatinib	HER mutations/amplification	33	II	≥2L	51.5%	6.9	6.0	14.8	G3 diarrhea (3%), G3 hypertension (9.1%)	[108]
**Monoclonal antibodies**											
IFCT 1703-R2D2	Pertuzumab Trastuzumab Docetaxel	HER2 alteration (exon-20 mutation or insertion)	47	II	≥2L	29%	6.8	11.0	17.6	64%	[109]
**Antibody–drug conjugates**											
Iwama et al.	TDM-1	HER2 mutation (exon-20 insertion)	22	II	≥2L	38.1%	2.8	3.5	8.1	22.7%	[110]
Peters et al.	TDM-1	HER2 IHC ≥2+	49	II	≥2L	0% (IHC 2+) 20% (IHC 3+)	2.6 (IHC 2+) 2.7 (IHC 3+)	3.6	12.2 (IHC 2+) 15.3 (IHC 3+)	22%	[111]
DESTINY- Lung01 Cohort 1	T-DXd (6.4 mg/kg)	HER2 IHC 2, 3+	49	II	≥2L	24.5%	5.4	6.0	NR	73.5%	[112]
DESTINY- Lung01 Cohort 2	T-DXd (6.4 mg/kg)	HER2 mutation	91	II	≥2L	55%	8.2	9.3	17.8	46%	[113]
DESTINY- Lung02	T-DXd (5.4 mg/kg or 6.4 mg/kg)	HER2 mutation	152	II	≥2L	49.0% (5.4 mg/kg) 56.0% (6.4 mg/kg)	9.9 (5.4 mg/kg) 15.4 (6.4 mg/kg)	16.8 (5.4 mg/kg) NR (6.4 mg/kg)	19.5 (5.4 mg/kg) NR (6.4 mg/kg)	31.7% (5.4 mg/kg) 58% (6.4 mg/kg)	[114]

ORR: objective response rate; mPFS: median progression-free survival; mDoR: median duration of response; mOS: median overall survival; G3+ TRAE: grade 3 or higher treatment-related adverse events; NR: not reported; QD: once a day; BID: twice a day; TDM-1: trastuzumab–emtansine; IHC: immunohistochemistry; T-DXd: trastuzumab–deruxtecan.
